# On a growth model for complex networks capable of producing power-law out-degree distributions with wide range exponents

**DOI:** 10.1038/srep09067

**Published:** 2015-03-13

**Authors:** J. Esquivel-Gómez, P. D. Arjona-Villicaña, E. Stevens-Navarro, U. Pineda-Rico, R. E. Balderas-Navarro, J. Acosta-Elias

**Affiliations:** 1 Instituto de Investigación en Comunicación Óptica, Universidad Autónoma de San Luis Potosí (UASLP), México; 2 Facultad de Ciencias, Universidad Autónoma de San Luis Potosí (UASLP), México; 3 Facultad de Ingenieria, Universidad Autónoma de San Luis Potosí (UASLP), México

## Abstract

The out-degree distribution is one of the most reported topological properties to characterize real complex networks. This property describes the probability that a node in the network has a particular number of outgoing links. It has been found that in many real complex networks the out-degree has a behavior similar to a power-law distribution, therefore some network growth models have been proposed to approximate this behavior. This paper introduces a new growth model that allows to produce out-degree distributions that decay as a power-law with an exponent in the range from 1 to ∞.

Among the topological properties of real complex networks (*CN*), one of the most studied is the out-degree distribution. This property describes the probability that a node in the network has a particular number of outgoing links. It has been found that in many real *CN* the out-degree behaves as a power-law distribution (*P*(*k*) ∝ *k*^−*γ*^)^1^,^2^,^3^,^4^,^5^,^6^,^7^. In order to approximate this type of out-degree distribution, some growth models for *CN* have been proposed. For example, Dorogovtsev *et.al.*^8^ and Bollobás *et.al.*^9^ have each developed a model capable of producing out-degree distributions that decay as a power-law with exponent 
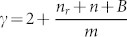
 and 
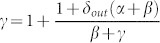
, respectively. Hence in both models the *γ* exponent is greater than 2. Esquivel *et.al.*^10^ proposed a model that produces out-degree distributions that decay as a power-law where the *γ* exponent value is in the range between 0 and 1.

The previous models are not able to produce out-degree distributions with *γ* exponents in the range between 1 and 2. However, there are real *CN* where the *γ* exponent value is within this interval. For example, the social network of Flickr users^6^, the Any Beat network^7^, the online social network Epinions^6^ and the network of flights between airports of the world (OpenFlights)^6^ where the *γ* exponent for the out-degree distribution of these *CN* is close to 1.74, 1.71, 1.69 and 1.74 respectively.

This paper introduces a new model for growth of directed *CN* that allows to obtain out-degree distributions that decay as a power-law with exponents in the range 1 < *γ* < ∞. That is, the proposed model is able to generate all exponent values found in documented real *CN*^1^,^2^,^3^,^4^,^5^,^6^,^7^.

It has been demonstrated that the growth and evolution of *CN* is influenced by local processes that shape its topological and dynamical properties^11^. The model proposed in here incorporates two local processes for adding new nodes to the network: a random out-degree selection and a copy of an already present out-degree value. In many large networks the maximum degree of a node (the degree of a node is the sum of its incoming and outgoing links) is much smaller than the number of nodes^6^. Thus, the proposed model assumes that the probability that a new node *n_new_* selects a random out-degree decreases as the network grows. This probability is expressed as *N*^−*α*^ where *N* is the total number of nodes in the network (including *n_new_*) and *α* is a constant greater than 0. In other words, the probability that new nodes have an out-degree close to *N* tends to zero as *N* ≫ 1.

## Proposed model

In this model, the growth of the network is performed by adding nodes one at a time. At the beginning, only node *n*_0_ is present in the network and its out-degree is 0. Then, the out-degree of any new node *n_new_* added to this network is determined as follows:

- With probability *N*^−*α*^, *n_new_* randomly selects an out-degree uniformly distributed from 0 to *N* − 1. That is, *n_new_* may have out-degree 0, 1, 2, …, *N* − 1. It is important to notice that it is possible that *n_new_* has an out-degree of the order of *N* − 1. In this situation *n_new_* would connect to all the other nodes in the network. This scenario may not be realistic for large values of *N*, because in many real networks, the maximum degree for a node is much smaller than the total number of nodes *N*^6^. However, the probability *N*^−*α*^ decreases when *N* increases for *α* > 0.
- With complementary probability 1 − *N*^−*α*^, *n_new_* copies the out-degree of a randomly selected node from the network. It is important to notice that as the number *Q_s_* of nodes with out-degree *s* increases, the probability that *n_new_* has out-degree *s* also increases to 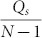.

It is possible to employ the continuum method^12^ to obtain the analytical solution for the proposed model. This method is implemented using the following differential equation:
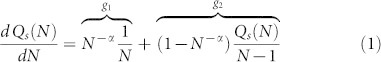
The previous equation describes the variation of the number *Q_s_* of nodes with out-degree *s* with respect to the total number *N* of nodes in the network. The term *g*_1_ describes the situation that a new node randomly selects an out-degree value and the term *g*_2_ the situation that a new node copies this value from a randomly selected node in the network.

**Figure 1:**
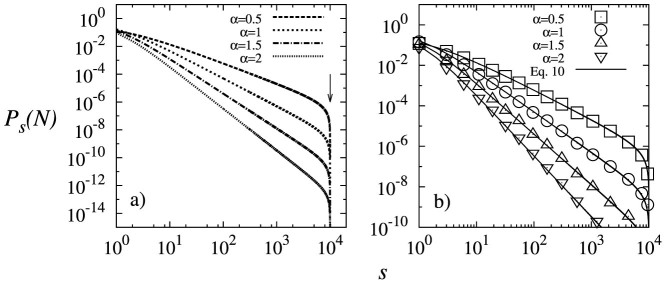
(a) Analytical solution of the proposed model (Eq. 10 in dashed lines) for *N* = 10^4^ and different *α* values. Notice that the proposed model is able to obtain out-degree distributions *P_s_* that decay as a power-law. Also, it may be noted that for values of *s* close to *N* the *P_s_* decays rapidly (vertical arrow) and the power-law behavior is cut-off. (b) Comparison of the out-degree distribution produced by the experiments (symbols ⊡, ⊙, △, ▽) and by Eq. 10 (solid line) for *N* = 10^4^ and several values of *α*.

Eq. 1 may be written in the standard form for a linear differential equation:
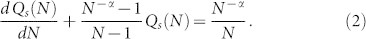
From Eq. 2, it is possible to deduce the integrating factor 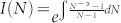. Solving for *I*(*N*) produces non elementary functions, which complicate the solution of Eq. 2. In order to obtain an integrating factor in terms of elementary functions, it is best to simplify Eq. 2 as follows:
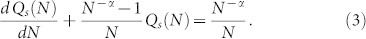


This simplification has little implications for large values of *N*, because *N* − 1 ≈ *N*, as *N* ≫ 1. This allows to employ the following integrating factor: 
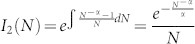
. Multiplying Eq. 3 by *I*_2_(*N*) produces:
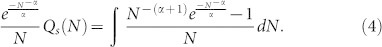
Solving for *Q_s_*(*N*)
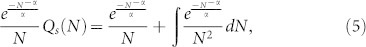

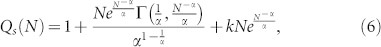
where *k* is a constant and Γ(·) is the incomplete Gamma function. In order to obtain the out-degree distribution *Q_s_*(*N*), it is necessary to solve Eq. 6 for *s* = 1, *s* = 2, and so on as follows:

- for *Q*_1_(*N*), consider the initial condition
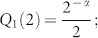
this initial condition is due to the fact that, at the beginning the network only has one node, *n*_0_, with no outgoing links (*N* = 1). When the next node, *n*_1_, is added (*N* = 2), the probability that node *n*_1_ has out-degree *s* = 1 is 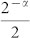.
- Then, solving Eq. 6 for the initial condition 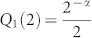 produces:
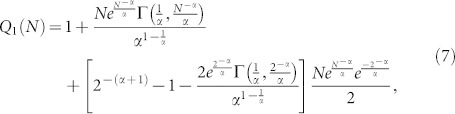
- for *Q*_2_(*N*), consider the initial condition
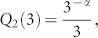
this initial condition is due to the fact that, before adding node *n*_2_ only *n*_0_ and *n*_1_ exist in the network (*N* = 2) and both have *s* < 2, therefore *Q*_2_(2) = 0. When *n*_2_ is added (*N* = 3), the probability that node *n*_2_ has out-degree *s* = 2 is 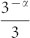.
- Then, solving Eq. 6 with the initial condition 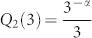, one obtains:
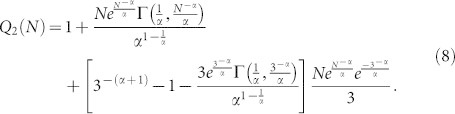

From the results in Eqs. 7 and 8, it is possible to deduce that:
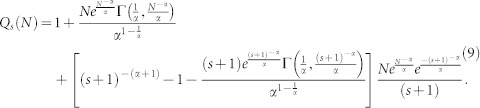


Normalizing Eq. 9, yields:
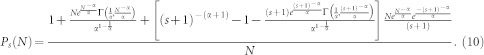


Eq. 10 describes the out-degree distribution *P_s_*(*N*) obtained with the proposed model for 1 < *s* < *N*. It can also be noted that, as *s* → *N*, Eq. 10 predicts that 
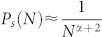
. That is *P_s_*(*N*) decays to 0 rapidly as *s* → *N* and *N* ≫ 1, therefore the power-law behavior exhibits a cut-off (Figure 1a).

In order to obtain the scaling exponent of the out-degree distribution, terms Γ(·) into Eq. 10 are simplified using:
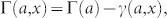
where *γ*(*a*, *x*) and Γ(*a*, *x*) are the lower and upper incomplete Gamma functions, respectively. By the following asymptotic property:
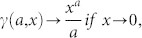
it is possible to write:
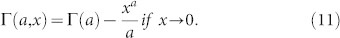


Using Eq. 11 it is possible rewrite the Γ(·) terms of Eq. 10 as follows:
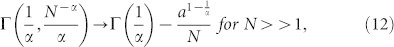

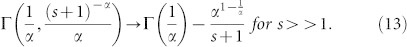


Substituting Eqs. 12 and 13 into Eq. 10 and considering that *s* + 1 ≈ *s* as *s* ≫ 1, Eq. 10 can be expressed as:
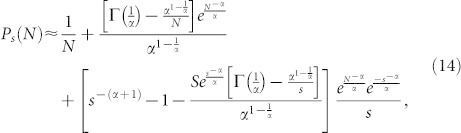

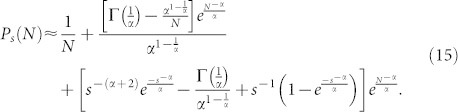


Using the two first terms of the series expansion of 
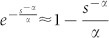
 in Eq. 15 and simplifying
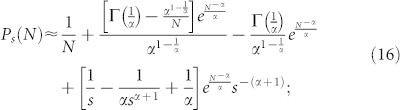
for *s* ≫ 1, 
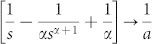
, thus it is possible to rewrite Eq. 16 as:
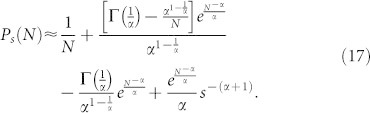


Furthermore, in the limit when *N* → ∞, Eq. 17 takes the form
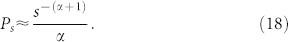


Eq. 18 shows that the out-degree distribution obtained with the proposed model decays as a power-law *P_s_* ~ *s*^−*γ*^ for 1 < *s* < *N* with scaling exponent *γ* = *α* + 1.

To validate the analytical solution of the model as described by Eq. 10, four experiments were executed using *α* = 0.5, 1, 1.5 and 2. Each of these experiments simulated the growth of a directed network from *N* = 1 to 10^4^ nodes. Figure 1b shows that the out-degree distribution produced by these experiments and the analytical predictions by Eq. 10 fit appropriately.

## Comparison with real networks

To verify that the proposed model is able to reproduce the out-degree distribution of real *CN*, the social network of Flickr users^6^ was selected.

**Figure 2:**
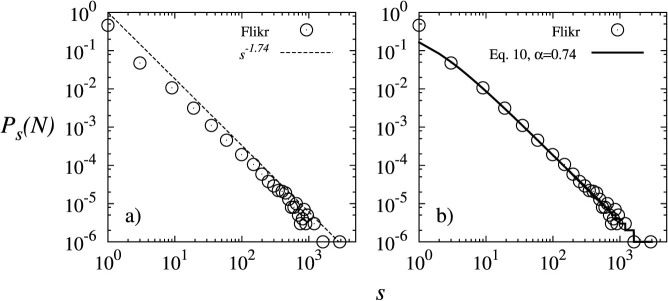
(a) Out-degree distribution of the Flickr social network. (b) Comparison of the out-degree distribution produced by the proposed model (Eq. 10) with *α* = 0.74 and *N* = 2, 302, 925 and the actual out-degree distribution of the Flickr social network.

In this network, the users correspond to the nodes and their friendship connections to the links. This network has 2, 302, 925 nodes and 33, 140, 017 links. Figure 2a shows that the out-degree distribution of the nodes in the Flickr network decay as a power-law distribution with *γ* ≈ 1.74. Figure 2b shows that the model proposed by Eq. 10 with *α* = 0.74 and *N* = 2, 302, 925 reproduces appropriately the out-degree distribution of the Flickr network for *s* > 1.

## Discussion

The model proposed in this article has been able to reproduce the out-degree distribution of the Flickr social network for values of *s* > 1. Although this model produces a good fit with the out-degree distribution of a real network, we cannot guarantee that the local processes incorporated in this model are the only ones involved in the behavior of the out-degree distribution of the nodes in this network. Unknown processes may help to explain why for *s* = 1, this model does not fit. However, the proposed model provides a simplification of these processes and therefore, reproduces the out-degree distribution of the network.

## Conclusions

Local processes participate in the growth and evolution of real *CN* which, in turn, shape the out-degree of its nodes. The model proposed here incorporates two local processes: a random out-degree selection and a copy of an out-degree for the nodes added to the network. This model is able to produce out-degree distributions that decay as a power-law with the *γ* exponent in the range from 1 to ∞. That is, the proposed model reproduces all exponent values found in distributions of documented real complex networks.

## Author Contributions

J.E.G., P.D.A.V., E.S.N., U.P.R., R.E.B.N. and J.A.E. contributed to this research and helped to edit this manuscript.
